# Acupuncture for chronic pelvic pain in patients with SPID: A protocol for systematic review and meta-analysis

**DOI:** 10.1097/MD.0000000000023916

**Published:** 2021-01-29

**Authors:** Tao Peng, Yang Wu, Li Huang, Bisong He, Shaobin Wei

**Affiliations:** Department of Gynecology, Hospital of Chengdu University of Traditional Chinese Medicine, No. 39 Shierqiao Road, Chengdu, Sichuan, China.

**Keywords:** acupuncture, chronic pelvic pain, meta-analysis, protocol, sequelae of pelvic inflammatory disease, systematic review

## Abstract

**Background:**

Chronic pelvic pain (CPP) is one of the common sequela of pelvic inflammatory disease, the pathological factors are adhesions, scarring and pelvic congestion which caused by inflammation, often cause abdominal pain and lumbosacral soreness, and aggravated after fatigue, sexual intercourse and during menstruation. It is difficult to treat because special pathological changes. Although acupuncture has gained increased popularity for the management of CPP, evidence regarding its efficacy is lacking. Therefore, a systematic review of acupuncture for chronic pelvic pain in patients with SPID is required to provide available evidence for further study.

**Methods and analysis:**

We will conduct a systematic review of randomized controlled trials (RCTs) that investigate the effect and safety of acupuncture for the treatment of chronic pelvic pain patients with SPID. We will electronically search the literature in the databases of PubMed, the Cochrane Central Register of Controlled Trials (CENTRAL), EMBASE, the Web of Science, China National Knowledge Infrastructure (CNKI), Wan-fang Digital Periodicals, Chinese Biomedical Literature Database (CBM), Chinese Scientific Journal Database (VIP) and select eligible articles. Data extraction will be conducted by 2 researchers independently, and risk of bias of the meta-analysis will be evaluated based on the Cochrane Handbook for Systematic Reviews of Interventions. The primary outcomes will be total effective rate and VAS pain score, and the secondary outcomes include the recurrence rate and adverse reaction. All data analysis will be conducted by software Review Manager V.5.3.

**Results:**

This study will provide the latest analysis of the currently available evidence for the efficacy of acupuncture for chronic pelvic pain in patients with SPID.

**PROSPERO registration number:**

CRD42020193826.

## 1 Introduction

Pelvic inflammatory disease (PID) is a group of infectious diseases of the female upper reproductive tract which ascending infection from the lower genital tract,^[1]^ including endometritis, salpingitis, tubo-ovarian abscess, peritonitis.^[2]^ Because of the signs and symptoms of PID are mild or are asymptomatic, many women with PID couldn’t be diagnosis and treatment in time probably leads to inflammatory sequelae in the upper genital tract.^[3]^ Sequelae of pelvic inflammatory disease (SPID) is the legacy lesions of PID, including infertility, ectopic pregnancy, chronic pelvic pain (CPP), PID recurrence long-term complications.^[4]^ It has been reported that 20% of acute pelvic inflammatory attacks will leave chronic pelvic pain. CPP is persistant, situationally, chronically pain has been continuous for at least 6 months.^[5]^ Its clinical manifestation is pelvic pain, abdominal pain, or lumbosacral pain.^[6]^ Apart from daily pain, symptoms such as painful intercourse, painful bowel motions and exacerbation of period pain are commonly reported by patients.^[7]^ It also can lead to certain psychological problems and sexual dysfunction.^[8]^ In women who suffering from CPP, often accompanied by anxiety, substance abuse or mental disorders.^[9,10]^ Western medicine has limited methods for treating CPP because of special pathological changes: adhesions, scarring, and pelvic congestion.

At present, the treatments of CPP are drug, surgery and psychotherapeutic.^[11,12]^ However, it is not sensitive to antibiotic treatment because CPP is often not a single disease entity rather a constellation of symptoms that can arise from other gynecology diseases.^[13]^ (e.g., PID, endometriosis, adenomyosis, pelvic varicosis.). The development of laparoscopy has opened up a new direction for the diagnosis and treatment of pelvic pain, laparoscopic adhesiolysis can relieve pelvic and abdominal pain. But there are also some surgical complications associated with the procedure such as intestinal injury.^[14,15]^ There is consistent scientific evidence showed laparoscopic adhesiolysis is not recommended for the management of chronic pelvic pain.^[16]^

In recent years, more and more attention has been paid to Traditional Chinese medicine (TCM) with better curative effect and less adverse reactions. Acupuncture is an important part in TCM which prevent and treat diseases. Since it is efficiently, simple and low cost, it has been widely used in China and countries around China for thousands of years, such as Korea and Japan. Acupuncture prevent and treat diseases by stimulating certain acupoints on the body to active the meridians and collaterals and to regulate the function of the internal organs, *qi* and blood. Just As *Huangdi Neijing* said: stagnation leading to pain. In the theory of TCM, *qi* is the leader of blood, *qi* can propel normal circulation of blood while stagnation of *qi* leads to blood stasis. Acupuncture can regulate *qi* and blood, dredge channels and collaterals, improve local blood circulation, improve blood viscosity, and thus reduce pain. There is an international consensus on acupuncture for analgesia, acupuncture has been extensively used by clinicians for the management of pain.^[17,18]^ A team led by Maiken Nedergaard of the university of rochester medical center reported their experimental results that during acupuncture, the body released adenosin on the adenosine A1 receptor by releasing adenosine and play the analgesic effect.^[19]^ It has been reported that acupuncture can play an anti-inflammatory role by participating in the regulation of inflammatory mediators in chronic inflammatory diseases through a variety of ways.^[20,21]^ Therefore, this study aims to evaluate the efficacy and safety of acupuncture for CPP patients with SPID through a systematic review and meta-analysis.

## 2 Methods

### 2.1 Study registration

This systematic review protocol has been registered in the International Prospective Register of Systematic Reviews (PROSPERO) and registration number CRD42020193826. The systematic review protocol followed the Preferred Reporting Items for Systematic Reviews and Meta-Analysis (PRISMA) Protocols guidelines and the Cochrane Handbook for Systematic Reviews of Interventions.^[22,23]^

### 2.2 Inclusion criteria for study selection

#### 2.2.1 Types of studies

All randomized controlled trials (RCTs) evaluating the efficacy of acupuncture for chronic pelvic pain in patients with SPID will be included. There will be no limit on the language and date publication. The animal mechanism studies, case reports, self pre-and post-control, or non-RCTs will be excluded.

#### 2.2.2 Types of participants

Patients is accord the diagnose criteria of CPID or SPID, and intermittent or constant pain in the abdomen or pelvis at least 3 months duration, not limited to the period of menstruation.

#### 2.2.3 Types of interventions and comparisons

We will include studies using acupuncture as an experimental intervention. Such as electro-acupuncture, warm needling, fire needling, body acupuncture, auricular acupuncture, scalp acupuncture, and plum blossom needle. Trials evaluating acupuncture combination with western medicine, sham acupuncture and no treatment for a control group will be included. We will exclude trials comparing the efficacy of different acupuncture therapies and trials proved the combined effect of acupuncture and other treatments (e.g., acupuncture with herbal, acupuncture with Chinese medicine enema, etc.). We will consider the following treatment comparisons.

1. Acupuncture vs western medicine.
2. Acupuncture vs sham acupuncture.
3. Acupuncture vs no treatment.
4. Acupuncture combined western medicine vs western medicine alone.

#### 2.2.4 Types of outcome measures

##### 2.2.4.1 Primary outcomes measures

The primary outcomes will include total effective rate and VAS pain score.

##### 2.2.4.2 Secondary outcomes measures

Include the recurrence rate and adverse reaction, such as pneumothorax, bleeding and infection.

### 2.3 Search strategy

#### 2.3.1 Electronic searches

We will electronically search the following databases from respective inceptions: PubMed, the Cochrane Central of Controlled Trials (CENTRAL), EMBASE, the Web of Science, China National Knowledge Infrastructure (CNKI), Wan-fang Digital Periodicals, Chinese Biomedical Literature Database (CBM), Chinese Scientific Journal Database (VIP). There is no language and date publication restriction. To fully search eligible studies, we will adopt a comprehensive retrieval strategy which combing with Mesh terms, text words, titles and synonyms. The search strategies for PubMed are summarized in Table 1. These search terms will be equivalent translated and apply to other databases.

**Table 1:** PubMed search strategy.

Number	Search strategy
1	Randomized controlled trial.pt
2	Controlled clinical trial.pt
3	Randomized. ti, ab
4	RCT. ti, ab
5	Placebo. ti, ab
6	Clinical Trials. ti, ab
7	Or 1–6
8	Pelvic pain. ti, ab
9	Chronic pelvic pain. Mesh
10	Pelvic inflammatory disease. ti, ab
11	Chronic pelvic pain. ti, ab
12	Chronic pelvic inflammatory disease. ti, ab
13	Sequelae of pelvic inflammatory disease. ti, ab
14	PID. ti, ab
15	CPP. ti, ab
16	SPID. ti, ab
17	Or 8–16
18	Acupuncture therapy. Mesh
19	Acupuncture. ti, ab
20	Acupoint. ti, ab
21	Electro-acupuncture. ti, ab
22	Warm needling. ti, ab
23	Fire needling. ti, ab
24	Scalp acupuncture. ti, ab
25	Manual acupuncture. ti, ab
26	Auricular acupuncture. ti, ab
27	Ear acupuncture. ti, ab
28	Plum blossom needle. ti, ab
29	Dermal needle. ti, ab
30	Body acupuncture. ti, ab
31	Or 18–30
32	7 and 17 and 31

### 2.4 Data collection and analysis

#### 2.4.1 Studies selection

**Figure 1:**
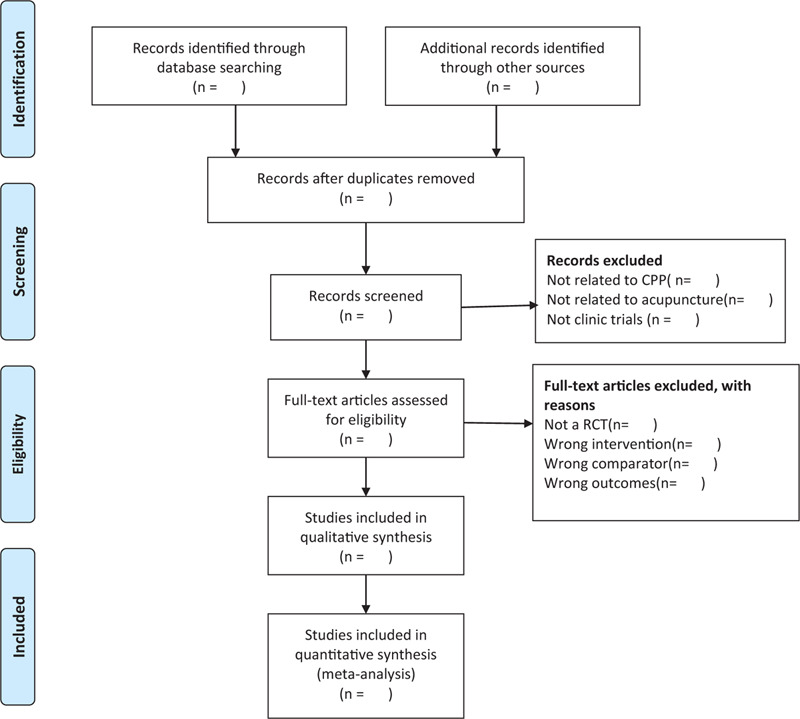
The PRISMA flow diagram of study selection process.

The selection of research study will be independently carried out by 2 researchers. Firstly, we will use EndNote X9 software to removing duplications and manage records. Secondly, 2 review authors (BSH and YW) independently screen titles and abstracts according to inclusion criteria and exclude any studies that are obviously not eligible. Then, 2 reviewers will obtain full-text of all relevant studies and independently and carefully screen the studies according to the inclusion criteria. Disagreement will be solved through discussion to reach a consensus. If it fails to reach a consensus by discussion, arbitrated by the third author (LH). The selection process is showed in the flow chart (Fig. 1).

#### 2.4.2 Data extraction and management

Two authors (LH and YW) will independently extract data according to the Cochrane Handbook for Systematic Reviews of Interventions. If 2 researchers have disagreement and cannot reach an agreement through discussion, reviewer (TP) will arbitrate. The data extraction form including 5 main elements:

1. General information: title, author, country, date of publication, language.
2. Methods: type of study, sample size, study duration, method of randomization, allocation concealment, blinding.
3. Participants characteristics: age, diagnostic criteria, disease duration.
4. Intervention: including observation group (types of acupuncture, acupuncture points, treatment duration, frequency) and control group.
5. Outcomes: primary and secondary outcomes, method of outcome assessments. For any missing data or unclear information we will try to contact the corresponding author.

#### 2.4.3 Assessment of risk of bias

We will use the Cochrane Collaboration's risk-of-bias tool assess the included studies bias. Two authors (TP and YW) will independently assess the risk of bias. The disagreement will be discussed to reach an agreement. If necessary we will consult a third review author (LH). Risk of bias assessment categories will include the following 7 domains:

1. randomized sequence generation;
2. allocation concealment;
3. blinding of participants and personnel;
4. blinding of outcome assessment;
5. incomplete outcome data;
6. selective outcome reporting;
7. other generic sources of biases.

#### 2.4.4 Measures of treatment effect

Meta-analysis will be conducted using Rev Man 5.3 software. Dichotomous variable will using the relative risk (RR) with 95% confidence intervals (CIs) as the measurable indicators to assess the treatment effect. For continuous variable, when the outcome scale is same, we will use the mean difference (MD) with 95%CIs to evaluate the treatment effect. If the same outcome variables using different methods, we will use the standardized mean difference (SMD) with 95%CIs to evaluate the treatment effect.

#### 2.4.5 Dealing with missing data

We will first contact the corresponding author or the first author if there is missing or insufficient data. If the missing data cannot be obtained, it will not be included in the analysis.

#### 2.4.6 Assessment of heterogeneity

The heterogeneity include clinical heterogeneity and methodological heterogeneity. We will use the χ^*2*^ test evaluate the heterogeneity of studies, with a *P* value of <.1 to be determine statistical significance. When the *I*^*2*^ value ≤50%, there is no heterogeneity among the studies. *I*^*2*^ value >50% will show there is heterogeneity in the included studies. We will qualitatively describe the effectiveness and safety of acupuncture if *I*^*2*^ value >75%.

#### 2.4.7 Assessment of reporting biases

When there are at least 10 studies included in the meta-analysis, we will create a funnel plot to assess the publication bias. An Egger test will be used to evaluate the funnel plot asymmetry.

#### 2.4.8 Data synthesis

Meta-analysis will be conducted using Rev Man V.5.3. According to heterogeneity if *I*^2^ value ≤50% we will select the fixed effects model, and if *I*^2^ value >50% random effects model will be selected. If *I*^2^ value >75%, we will just conduct a descriptive analysis.

#### 2.4.9 Subgroup analysis

If necessary we will conduct subgroup analysis to search the heterogeneity. Consider the following subgroup analysis plans.

1. Different types of intervention (acupuncture alone or combine with western medicine).
2. Different types of acupuncture (body acupuncture, electro-acupuncture, warm needling, etc.).
3. Duration of follow-up (1–3 months, up to 6 months, more than 6 months).

#### 2.4.10 Sensitivity analysis

We will conduct a sensitivity analysis to prove the stability of the research results. This will be achieved by removing small sample size studies and assessing the impact of risk of bias, missing data.

#### 2.4.11 Ethics and dissemination

There is no patient participate in this research, the basic of this research is published evidence. Therefore, ethics examination and agreement not needed. We aim to publish our findings to a peer-reviewed journal or scientific conferences.

## 3 Discussion

CPP is a common complication of PID in women.^[24]^ The limitations of Western medicine treatment of CPP because of its special pathological changes. The effectiveness and safety of acupuncture in reducing the pain of CPP have confirmed by many clinical and trial studies. However, there is no a systematic review aims to assess the efficacy and safety of acupuncture for CPP patients with SPID. This systematic review will provide latest convincing conclusions evidence-based basis for clinical acupuncture treatment for CPP patients with SPID. In addition, there are some possible limitations in this systematic review and meta-analysis. For example different types of acupuncture therapies may lead to the risk of heterogeneity, it is difficult to obtain the complete primary data from original trials, etc. We hope this systematic review and meta-analysis will provide the latest analysis of the evidence for the efficacy of acupuncture in treating CPP, which will benefit clinicians and patients.

## Author contributions

**Investigation:** Tao Peng, Shaobin Wei.

**Methodology:** Yang Wu.

**Resources:** Tao Peng, Li Huang, Yang Wu, Bisong He.

**Supervision:** Shaobin Wei.

**Writing – original draft:** Tao Peng.

**Writing – review & editing:** Shaobin Wei.

## Abbreviations

Abbreviations: CPP = chronic pelvic pain, PID = pelvic inflammatory disease, RCTs = randomized controlled trials, SPID = sequelae of pelvic inflammatory disease, TCM = traditional Chinese medicine.
